# Tris(1,3-dichloropropyl) phosphate – Determination of bis(1,3-dichloropropyl) phosphate in urine by LC-APCI-ESI-MS/MS: Biomonitoring Method – Translation of the German version from 2024

**DOI:** 10.34865/bi72236e9_4or

**Published:** 2024-12-23

**Authors:** Petra Krystek, Henry Beeltje, Marc Machiel George Houtzager, Eric Martin van den Hoeven, Laura Kuhlmann, Elisabeth Eckert, Thomas Göen, Andrea Hartwig, MAK Commission

**Affiliations:** 1 TNO – Location Utrecht. Environmental Monitoring. Sensing and Analysis P.O. Box 80015 NL-3508 TA Utrecht The Netherlands; 2 Friedrich-Alexander-Universität Erlangen-Nürnberg. Institute and Outpatient Clinic of Occupational, Social, and Environmental Medicine Henkestraße 9–11 91054 Erlangen Germany; 3 Institute of Applied Biosciences. Department of Food Chemistry and Toxicology. Karlsruhe Institute of Technology (KIT) Adenauerring 20a, Building 50.41 76131 Karlsruhe Germany; 4 Permanent Senate Commission for the Investigation of Health Hazards of Chemical Compounds in the Work Area. Deutsche Forschungsgemeinschaft, Kennedyallee 40, 53175 Bonn, Germany. Further information: Permanent Senate Commission for the Investigation of Health Hazards of Chemical Compounds in the Work Area | DFG

**Keywords:** TDCPP, BDCPP, flame retardant, biomonitoring, urine, LC-APCI-ESI-MS/MS

## Abstract

The working group “Analyses in Biological Materials” of the German Senate Commission for the Investigation of Health Hazards of Chemical Compounds in the Work Area (MAK Commission) developed and verified this biomonitoring method for the determination of urinary concentrations of bis(1,3-dichloropropyl) phosphate (BDCPP), which is the major metabolite of tris(1,3-dichloropropyl) phosphate (TDCPP). TDCPP is one of the most commonly used organophosphate flame retardants in cars, residential furniture, and products containing polyurethane foam, and has been detected in dust from private houses, office buildings, and car interiors, suggesting that a majority of the general population is exposed to TDCPP. The aim of this work was to establish a reliable, selective, and sensitive method for the detection of BDCPP in urine. The urine samples are spiked with the internal standard d_10_-BDCPP and slightly acidified. Cleanup by mixed-mode anion-exchange solid-phase extraction is applied, and BDCPP is detected by liquid chromatography with simultaneous atmospheric pressure chemical ionisation and electrospray ionisation-tandem mass spectrometry. Calibration is carried out in ultra-pure water ∶ MeOH (4 ∶ 1, v/v). Good precision data with standard deviations below 7%, as well as good accuracy data with mean relative recoveries in the range of 93.6–101%, show that the method provides reliable and accurate analytical results. The method is both selective and sensitive, and the limit of quantitation of 0.2 ng BDCPP/l urine is sufficient to determine occupational exposure as well as higher background exposure levels to TDCPP in the general population.

## 1 Characteristics of the method

**Table:** 

**Matrix**		Urine	
**Analytical principle**		Liquid chromatography with simultaneous atmospheric pressure chemical ionisation and electrospray ionisation-tandem mass spectrometry (LC‑APCI‑ESI‑MS/MS)	
**Parameter and corresponding hazardous substance**			
**Hazardous substance**	**CAS No.**	**Parameter**	**CAS No.**
Tris(1,3‑dichloropropyl) phosphate (TDCPP)	13674-87-8	Bis(1,3‑dichloropropyl) phosphate (BDCPP)	72236-72-7

### Reliability data

#### BDCPP

**Table:** 

Within-day precision:	Standard deviation (rel.)	*s*_*w*_ = 6.5%, 3.1%, or 5.5%
	Prognostic range	*u* = 15.4%, 7.3%, or 13.0%
	at a spiked concentration of 0.9 μg, 9 μg, or 90 μg BDCPP per litre of urine and n = 8 determinations	
Day-to-day precision:	Standard deviation (rel.)	*s*_*w*_ = 8.5%, 8.7%, or 5.5%
	Prognostic range	*u* = 20.1%, 20.6%, or 13.0%
	at a spiked concentration of 0.9 μg, 9 μg, or 90 μg BDCPP per litre of urine and n = 8 determinations	
Accuracy:	Recovery (rel.)	*r* = 93.6%, 99.5%, or 100.5%
	at a spiked concentration of 0.9 μg, 9 μg, or 90 μg BDCPP per litre of urine and n = 8 determinations	
Limit of detection:	0.06 μg BDCPP per litre of urine	
Limit of quantitation:	0.2 μg BDCPP per litre of urine	

## 2 General information on TDCPP

Tris(1,3‑dichloropropyl) phosphate (TDCPP) is one of the most commonly used organophosphate flame retardants (OPFRs) in flexible foams for the automotive industry. It is liquid at room temperature and exhibits a low vapour pressure (5.6 × 10^−6^ Pa at 25 °C) (OECD 2009). The typical mass fraction of TDCPP in polyurethane foam, for example, is in the range of 5–10 wt.%. A smaller but still significant amount of TDCPP is used in flexible foams for residential furniture (van der Veen and de Boer 2012). As TDCPP is an additive-type flame retardant, it may partially diffuse out of the treated substrate.

Recent studies have detected TDCPP in dust from private homes, office buildings, and automobiles, suggesting that a large portion of the general population is chronically exposed to TDCPP. Occupational exposure can occur in the production of TDCPP, in the production and processing of flexible and composite foams, and in the manufacture of automotive parts (EU 2008). Firefighters may be exposed to TDCPP as well (Jayatilaka et al. 2017).

In case of TDCPP exposure, different routes of exposure are of relevance (Carignan et al. 2013; Norén et al. 2017). In an unpublished *in vitro* dermal-absorption study, human-skin membranes were exposed to ^14^C‑TDCPP for 8 hours, simulating a normal workday. The mean total absorption was 15.4%, 10.69%, and 6.0% for applied doses of 0.003 mg, 0.01 mg, and 0.12 mg TDCPP/cm² skin, respectively (EU 2008). After exposure via oral or inhalation routes, 100% absorption can be assumed (EU 2008). Kinetic and distribution studies in rats have, however, demonstrated that TDCPP is rapidly metabolised and excreted, such that no significant accumulation in the body is to be expected (Krystek et al. 2019).

A study in rats by Nomeir et al. (1981) showed that the uptake route of radiolabelled TDCPP had little effect on its distribution in the body. Moreover, the gastrointestinal absorption and distribution of TDCPP were unaffected over a dosage range of two orders of magnitude. ^14^C‑TDCPP was rapidly metabolised and, within the first 24 hours, more than 80% of the radioactivity was excreted with the urine or faeces or was exhaled as ^14^CO_2_. The major metabolite eliminated with the urine was bis(1,3‑dichloropropyl) phosphate (BDCPP) (Nomeir et al. 1981). At the same time, Lynn et al. (1981) also investigated the distribution, metabolism, and excretion of TDCPP in rats. Five days after intrave­nous administration of ^14^C‑TDCPP, 92% of the administered dose were excreted with the urine (54%), faeces (16%), and exhaled air (22%, as ^14^CO_2_); another 4% were recovered in the body. The major urinary, faecal, and biliary metabolite was identified as BDCPP (Lynn et al. 1981). In a further TDCPP metabolism study in human-liver fractions *in vitro*and in rats *in vivo*, BDCPP was likewise confirmed as the main metabolite (Van den Eede et al. 2013 a).

**Fig.1:**
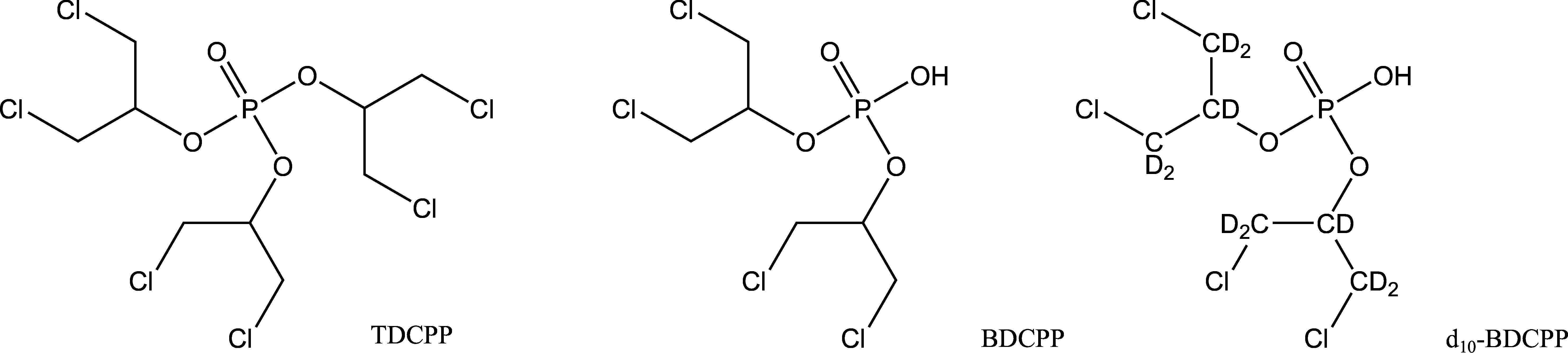
Structural formulas of TDCPP and BDCPP as well as the internal standard d_10_‑BDCPP

As no other known OPFR is metabolised to BDCPP (EU 2008), BDCPP is the most appropriate metabolite for the human biomonitoring of TDCPP (Figure 1)*. *According to the harmonised classification and labelling, this substance is suspected of causing cancer (European Commission 2018). TDCPP has not yet been evaluated by the Commission.Urinary BDCPP concentrations from the general population as well as in urine from potentially exposed workers, as published in the literature, are shown in Tables 1 and 2.

**Tab.1:** BDCPP in urine from the occupationally non-exposed general population

Study collective (country; age; n)	LOD [μg/l]	LOQ [μg/l]	BDCPP [μg/l]		References
			Mean ± SD	Range	
Adults (Belgium; age n. s.; 14)	0.04	0.15	0.29 ± 0.27 0.19 (median)	0.06–0.90 (DF = 100)	Bastiaensen et al. 2018
Adults (Belgium; 20–50 a.; 10)	–	0.05	0.11 (median)^a)^; 0.19 (median)^b)^	0.03–4.58^a)^(DF =66,3)^a)^; 0.06–10.79^b)^(DF = 100)^b)^	Bastiaensen et al. 2021
Adult women (USA; 18–46 a; 31^c)^)	0.056	–	0.83 (GM)^d)^	0.06–9.87 (DF = 100)	Carignan et al. 2017
Adult women (USA; 18–46 a; 30^e)^)			0.69 (GM)^d)^	< LOD–10.38 (DF = 97)	
Adults (Taiwan; >17 a; 317)	–	0.02	0.69	0.48–0.89^f)^(DF = 90)	Cheng et al. 2023
Adults (USA; 23–46 a; 9)	0.008	–	0.148 (GM) 0.083 (median)	0.046–1.662 (DF = 100)	Cooper et al. 2011
Adults (USA; age n. s.; 16)	0.02	–	0.46 0.09 (median)	< LOD–3.9 (DF = 94)	Dodson et al. 2014
Adults (USA; 19–67 a; 53)	–	–	0.37 (GM)^d)^	< LOD–4.46 (DF = 83.0)	Hoffman et al. 2015
Adult men with fertility problems (USA; 18–54 a; 355 samples from 220 men)	0.00002–0.00011	–	0.62 (GM)^d)^	< LOD–10.3 (DF = 85.1)	Ingle et al. 2018
Adults (USA; age n. s.; 76)	0.11	–	0.69 (median)	0.31–6.8 (DF = 100)	Jayatilaka et al. 2017
Adults (Netherlands; age n. s.; 40)	0.06	0.2	0.14	< LOD–0.66 (DF = 33.3)	Krystek et al. 2019
Adults (China; 20–80 a; 1863)	0.06	–	0.10 (median)^d)^	< LOD–0.23 (DF = 60.7)	Liu et al. 2024
Adult men (USA; 28–46 a; 33); urine collected in the afternoon	0.0327	–	0.384 (GM)	(DF = 100)	Meeker et al. 2013 a
Adult men (USA; 28–46 a; 33); urine collected in the morning			0.122 (GM)		
Adult men (USA; 18–54 a; 45)	0.0327	–	0.13 (GM) 0.12 (median)	< LOD–25.0 (DF = 91)	Meeker et al. 2013 b
Inuit adults (Canada; > 16 a; 28; sampling in 2017)	–	0.05	0.53 0.49 (GM) 0.45 (median)	0.27–1.70 (DF = 100)	Nero et al. 2024
Inuit adults (Canada; > 16 a; 1367; sampling in 2018–2019)			1.1 0.46 (GM) 0.49 (median)	< LOD–4.00 (DF = 94.4)	
Adults (USA; age n. s.; 13)	0.025	0.1	3.4 ± 2.5 2.5 (GM) 2.4 (median)	0.5–7.3 (DF = 100)	Petropoulou et al. 2016
Adult women (Canada; 18–45 a; 117)	0.21	0.52	2.4^g)^	< LOQ–26.3 (DF = 75)	Siddique et al. 2020
Adults (Vietnam; 18–76 a; 42)	0.02	0.105	11.9^g)^ 2.65 (GM)^g)^	0.03–49.6 (DF = 50)	Trinh et al. 2024
Adults (Belgium; Ø 40.8 a; 59)	–	0.52	–	< LOD–15 (DF = 15)	Van den Eede et al. 2013 b
Adults (USA; 33.8 ± 12 a; 213 samples from 19 individuals)	0.0102	–	0.737 0.414 (GM) 0.359 (median)	0.021–5.650 (DF = 100)	Wang et al. 2019
Adults (China; 44.62 ± 10.58 a; 1981)	0.064	–	0.12 (GM)^d)^ 0.12 (Median)^d)^	< LOQ–0.22 (DF = 60.9)	Xu et al. 2024

a: year; DF: detection frequency in % (percentage of measured values above LOD or LOQ); GM: geometric mean; LOD: limit of detection; LOQ: limit of quantitation; n: sample size; n. s.: not stated; SD: standard deviation

a) spot-urine samples (n = 309)

b) 24-h urine (n = 10)

c) measured as duplicates stored in glass and plastic vials

d) Summary statistics were calculated using LOD/√2 for values below the LOD.

e) measured as duplicates stored only in plastic vials

f) 95% confidence interval

g) Summary statistics were calculated using the LOD/2 for values below the LOD.

**Tab.2:** BDCPP in urine from potentially exposed workers

Study collective (country; age; n)	LOD [μg/l]	LOQ [μg/l]	BDCPP [μg/l]		References
			Mean ± SD	Range	
Female firefighters (USA; ≥ 18 a; 86)	0.2	–	4.08 (GM), 4.53 (GSD)^a)^	1.30–32.22 (DF = 100)	Trowbridge et al. 2022
Office workers (USA; ≥ 18 a; 84)			0.96 (GM), 3.99 (GSD)^a)^	< LOD–8.72 (DF = 90)	
Adult firefighters (USA; age n. s.; 146)	0.11	–	3.4 (median)	0.30–44 (DF = 100)	Jayatilaka et al. 2017
Controls, general population (USA; age n. s.; 76)			0.69 (median)	0.31–6.8 (DF = 100)	
Male firefighters (South Korea; 38–56 a; 149)	–	0.14	0.12^a)^ < LOQ (median)	< LOD–2.40^a)^(DF = 6.7)	Lim et al. 2023
Firefighters (USA; 21–52 a; 36)	0.11	–	2.38 μg/g crea (GM), 2.12 (GSD) pre-fire^a)^	(DF = 100)	Mayer et al. 2021
Workers in e-waste removal (China; 18–72 a; 30); morning urine	–	0.20	0.58 (median)	< LOD–18.5 (DF = 80)	Qin et al. 2021
Workers in e-waste removal (China; 18–72 a; 30); evening urine			0.53 (median)	< LOD–20.4 (DF = 93)	
Workers in e-waste recycling (China; 29–64 a; 42): morning urine on Day 3	–	0.02	1.66 ± 2.78 0.89 (median)	< LOD–12.8 (DF = 91)	Shi et al. 2019
Workers in e-waste recycling (China; 29–64 a; 42); evening urine on Day 3			1.03 ± 1.13 0.53 (median)	< LOD–3.91 (DF = 87)	
Hotel employees (China; 23–57 a; 26)	0.064	0.21	0.19 (GM) 0.21 (median)	< LOQ–2.1 (DF = 79)	Tao et al. 2018
Waste-incineration workers (China, 28.2 ± 4.3 a; 73)	0.12	–	0.18 (median)^b)^	< LOD–1.75 (DF = 82.2)	Wu et al. 2023
Controls, general population (China, 27.4 ± 5.1 a; 97)			0.06 (median)^b)^	< LOD–0.61 (DF = 11.3)	
Workers in e-waste recycling (China, age n. s.; 88)	–	0.03	0.23 (median)^b)^	< LOD–31.8 (DF = 82)	Yan et al. 2018
Waste-incineration workers (China, age n. s.; 30)			0.22 (median)^b)^	< LOD–3.56 (DF = 93)	

a: year; DF: detection frequency in % (percentage of measurement values above the LOD or LOQ); GM: geometric mean; GSD: geometric standard deviation; LOD: limit of detection; LOQ: limit of quantitation; n: sample size; n. s.: not stated; SD: standard deviation

a) Summary statistics were calculated using LOD/√2 for values below the LOD.

b) Summary statistics were calculated using LOD/2 for values below the LOD.

## 3 General principles

For the determination of BDCPP in urine, the diluted samples are slightly acidified and mixed with the internal stan­dard d_10_‑BDCPP. Afterwards, the samples are purified by solid-phase extraction using SPE cartridges (weak anion exchanger, polymeric). BDCPP is separated from matrix components by liquid chromatography and detected by tandem mass spectrometry with simultaneous atmospheric pressure chemical ionisation (APCI) and electrospray ionisation (ESI). External calibration is used for quantitative evaluation.

## 4 Equipment, chemicals, and solutions

### 4.1 Equipment

- LC‑MS/MS system (e.g. Agilent 6460 Triple Quad with a multimode source coupled to an Agilent 1200 HPLC system, Agilent Technologies, Inc., Santa Clara, CA, USA)
- Kinetex^®^LC column (2.6 μm biphenyl 100 Å, 100 × 2.1 mm) (e.g. No. 00D‑4622‑AN, Phenomenex Inc., Torrance, USA) with a UHPLC biphenyl precolumn with a 2.1‑mm ID (e.g. SecurityGuard ULTRA Cartridge No. AJ0‑9209, Phenomenex Inc., Torrance, CA, USA)
- Analytical balance (e.g. Sartorius AG, Göttingen, Germany)
- SPE vacuum manifold (e.g. IST VacMaster, Biotage Sweden AB, Uppsala, Sweden)
- Blow-off station (e.g. TurboVap^®^LV, Biotage Sweden AB, Uppsala, Sweden)
- SPE cartridges (weak anion exchanger, polymeric) (e.g. No. 8B‑S038‑UBJ, Strata‑X‑AW 33 μm, 60 mg/3 ml, Phenomenex Inc., Torrance, CA, USA)
- pH‑indicator strips pH 0–14 (e.g. No. 1.09535, MQuant^®^, Merck KGaA, Darmstadt, Germany)
- pH‑indicator strips pH 5–10 (e.g. No. 1.09533, MQuant^®^, Merck KGaA, Darmstadt, Germany)
- Syringe filters (nylon membrane, membrane diameter of 13 mm, pore size of 0.2 μm) (e.g. No. 6870‑1302, Whatman^®^GD/X^TM^, GE HealthCare Life Sciences, Buckinghamshire, United Kingdom)
- 10‑ml, 100‑ml and 1000‑ml volumetric flasks (e.g. BRAND GMBH + CO KG, Wertheim, Germany)
- 100‑ml wide-neck amber glass bottles with polypropylene screw caps for urine collection (e.g. No. 215‑4382, VWR International GmbH, Darmstadt, Germany)
- 100‑ml graduated cylinder (e.g. SCHOTT AG, Mainz, Germany)
- 15‑ml polypropylene tubes with screw caps (e.g. No. 525‑0604, VWR International GmbH, Darmstadt, Germany)
- 8‑ml threaded vials (e.g. No. 548‑0821A, VWR International GmbH, Darmstadt, Germany)
- 2‑ml screw-top glass vials and matching screw caps with PTFE/silicone septa (e.g. No. 5182‑0715 and No. 5185‑5820, Agilent Technologies Netherlands BV, Amstelveen, Netherlands)

### 4.2 Chemicals

Unless otherwise specified, all chemicals must be a minimum of *pro analysi*grade.

- BDCPP (e.g. No. TRC‑B419095‑10MG, Toronto Research Chemicals, Toronto, Canada)
- d_10_‑BDCPP (ISTD) (e.g. No. TRC‑B419097‑10MG, Toronto Research Chemicals, Toronto, Canada)
- Acetic acid, ≥ 99.7% (e.g. No. 695092, Fluka^TM^, Honeywell Deutschland Holding GmbH, Offenbach, Germany)
- Methanol, ≥ 99.8%, HiPerSolv CHROMANORM^®^for HPLC (e.g. No. 152505N, VWR International BV, Amsterdam, Netherlands)
- Acetonitrile, ultra-gradient, ultra-pure (e.g. No. 10614471, J. T. Baker Chemicals N. V., Deventer, Netherlands)
- Pyrrolidine (e.g. No. 807494, Merck KGaA, Darmstadt, Germany)
- Formic acid (e.g. No. 56302, Honeywell Specialty Chemicals Seelze GmbH, Seelze, Germany)
- Ultra-pure water (resistivity of 18.2 MΩ × cm) (e.g. from a central supply system)
- Nitrogen 5.0 (e.g. Air Liquide Deutschland GmbH, Düsseldorf, Germany)

### 4.3 Solutions

- Methanol ∶ water (1 ∶ 4, v/v) In a 100‑ml volumetric flask, 20 ml of methanol are mixed with 80 ml of ultra‑pure water.
- 5% pyrrolidine in acetonitrile 5 ml of pyrrolidine are pipetted into a 100‑ml volumetric flask. The flask is then made up to the mark with acetonitrile.
- Acetic acid (0.1 mol/l) 572 μl of acetic acid are pipetted into a 100‑ml volumetric flask containing some ultra-pure water. The flask is then made up to the mark with ultra-pure water.
- Eluent A (0.1% (v/v) formic acid in ultra-pure water) 1 ml of the concentrated formic acid is pipetted into a 1000‑ml volumetric flask. The flask is then made up to the mark with ultra-pure water.
- Eluent B (0.1% (v/v) formic acid in methanol) 1 ml of the concentrated formic acid is pipetted into a 1000‑ml volumetric flask. The flask is then made up to the mark with methanol.

### 4.4 Internal standard (ISTD)

- ISTD stock solution (1000 mg/l) 10 mg of the ISTD d_10_‑BDCPP are weighed exactly into a 10‑ml volumetric flask and dissolved in a little acetonitrile. The volumetric flask is subsequently made up to the mark with acetonitrile.
- ISTD working solution (10 mg/l) 100 μl of the ISTD stock solution are pipetted into a 10‑ml volumetric flask. The volumetric flask is subsequently made up to the mark with methanol.
- ISTD spiking solution (1 mg/l) 100 μl of the ISTD working solution are pipetted into a 2‑ml vial. Subsequently, 900 μl of methanol are added and the solution is mixed.

The ISTD stock and working solutions are stored in the freezer at −21 °C. The ISTD spiking solution must always be freshly prepared.

### 4.5 Calibration standards

- BDCPP stock solution (1000 mg/l) Approximately 10 mg of BDCPP are weighed exactly into a 10‑ml volumetric flask and dissolved in a little acetonitrile. The volumetric flask is then made up to the mark with acetonitrile.
- BDCPP working solution (10 mg/l) 100 μl of the BDCPP stock solution are pipetted into a 10‑ml volumetric flask. The volumetric flask is then made up to the mark with methanol.
- BDCPP spiking solution 1 (SL 1; 1 mg/l) 100 μl of the BDCPP working solution as well as 100 μl of the d_10_‑BDCPP working solution are pipetted into a 2‑ml vial. Subsequently, 800 μl of methanol are added and the solution is mixed.
- BDCPP spiking solution 2 (SL 2; 0.01 mg/l) 10 μl of SL 1 and 990 μl of methanol are pipetted into a 2‑ml vial and mixed.

The BDCPP stock and working solutions are stored in the freezer at −21 °C. The BDCPP spiking solutions must always be freshly prepared.

The calibration standards are prepared in 2‑ml vials according to the pipetting scheme given in Table 3. The concentration of the ISTD is always equal to the BDCPP concentration of the calibration standard. These calibration standards are processed analogously to the urine samples according to Section 5.2, without the addition of further ISTD, then analysed.

**Tab.3:** Pipetting scheme for the preparation of calibration standards for the determination of BDCPP in urine

Calibration standard	SL 2 [μl]	SL 1 [μl]	Methanol : water (1 : 4, v/v) [μl]	BDCPP [μg/l]	d_10_‑BDCPP [μg/l]
1	10	–	990	0.1	0.1
2	20	–	980	0.2	0.2
3	50	–	950	0.5	0.5
4	100	–	900	1	1
5	200	–	800	2	2
6	500	–	500	5	5
7	–	10	990	10	10
8	–	20	980	20	20
9	–	50	950	50	50
10	–	100	900	100	100

## 5 Specimen collection and sample preparation

### 5.1 Specimen collection

Urine samples are collected in pre-cleaned 100‑ml amber glass bottles. If the samples are not immediately processed, they must be cooled during transport and stored at −21 °C until analysis. Under these conditions, the urine samples are stable for at least one week (see Section 11.5).

### 5.2 Sample preparation

Frozen urine samples are thawed at room temperature and thoroughly mixed. 5 ml of urine are pipetted into a 15‑ml polypropylene vial and mixed with 10 μl of the ISTD spiking solution (1 mg d_10_‑BDCPP/l). After a 1 ∶ 1 (v/v) dilution with ultra-pure water, the pH value is adjusted to pH 6 with about 450 μl of diluted acetic acid (0.1 mol/l). The pH range is first verified using pH 0–14 test strips and a final verification of the correct pH adjustment is performed with pH 5–10 test strips. 

Solid-phase extraction (SPE) is then performed using Phenomenex StrataX AW cartridges. For this purpose, the cartridges are conditioned with 2 ml of methanol, followed by 2 ml of ultra-pure water. The urine samples are then applied to the cartridges (< 1 ml/min), washed with 2 ml of ultra-pure water, and dried under a vacuum. The analyte is eluated from the cartridges into 8‑ml glass vials using 2 ml of acetonitrile with 5% pyrrolidine. The eluated samples are evaporated to dryness at room temperature under a steam of nitrogen. Finally, the residue is reconstituted in 500 μl methanol ∶ water (1 ∶ 4, v/v) and filtered through a 0.2‑μm syringe filter into a 2‑ml HPLC vial.

## 6 Operational parameters

Analytical determination was performed on a device configuration comprised of an LC system and a tandem mass spectrometer (LC‑MS/MS). The developers of the method used a tandem mass spectrometer with a multimode source (Agilent 6460) and applied atmospheric pressure chemical ionisation simultaneously with electrospray ionisation. 

The adjustments described in this section are instrument-specific and must be tested and adapted by the user as needed. As such, the information given here is only intended as a point of reference. It may be necessary to make further adjustments to instrumentation from other manufacturers.

### 6.1 Liquid chromatography

**Table:** 

Analytical column:	Kinetex^®^core-shell silica (2.6 μm biphenyl 100 Å, 100 × 2.1 mm)
Precolumn:	UHPLC biphenyl, 2.1-mm ID
Separation principle:	Reversed phase
Column-oven temperature:	60 °C
Injection volume:	5 μl
Eluent:	A: 0.1% formic acid in ultra-pure water
	B: 0.1% formic acid in methanol
Runtime:	15 min
Gradient programme:	see Table 4

**Tab.4:** Gradient programme for the determination of BDCPP in urine

Time [min]	Eluent A [%]	Eluent B [%]	Flow rate [ml/min]	Pressure^a)^ [hPa]
0.0	90	10	0.500	600 000
10.0	10	90		
13.0	10	90		
13.1	90	10		
15.0	90	10		

a)  Initial pressure: 330 000 hPa at 90% Eluent A

### 6.2 Tandem mass spectrometry

**Table:** 

Ionisation:	Simultaneous atmospheric pressure chemical ionisation (APCI) and electrospray ionisation (ESI) in negative ionisation mode
Detection mode:	Multiple Reaction Monitoring (MRM)
Gas temperature (N_2_):	350 °C
Vaporiser temperature:	150 °C
Gas flow (N_2_):	5 ml/min
Nebuliser pressure:	60 psi
Capillary voltage:	−4000 V
Corona current:	1 μA
Multiplier:	500 delta EMV (+)
Parameter-specific settings:	see Table 5

The retention times, mass transitions, and further MS/MS parameters are given in Table 5. To reduce signal suppression, all urine samples were 1 ∶ 5 diluted prior to measurement.

**Tab.5:** Retention times, mass transitions, and MS/MS parameters for the determination of BDCPP in urine

Substance	Retention time [min]	Precursor ion (***m/z***)	Product ion (***m/z***)	Collision energy [V]	Cone voltage [V]	Cell-accelerator voltage [V]
BDCPP	5.97	319	35.1^a)^	16	60	5
		319	37.0^b)^	16	60	5
		318	35.1^b)^	8	80	5
d_10_‑BDCPP	5.89	329	35.1^a)^	12	80	5
		329	37.1^b)^	16	80	5
		328	35.0^b)^	8	80	5

a) Quantifier

b) Qualifier

## 7 Analytical determination

For the analytical determination of BDCPP, 5 μl of each of the urine samples processed according to Section 5.2 are injected into the LC‑MS/MS system and analysed under the conditions specified in Section 6. The analyte BDCPP is identified by its retention time and characteristic mass transitions.

The retention times given in Table 5 can only serve as a point of reference. The user of the method must ensure the separation performance of the used column and the resulting retention behaviour of the analyte.

**Fig.2:**
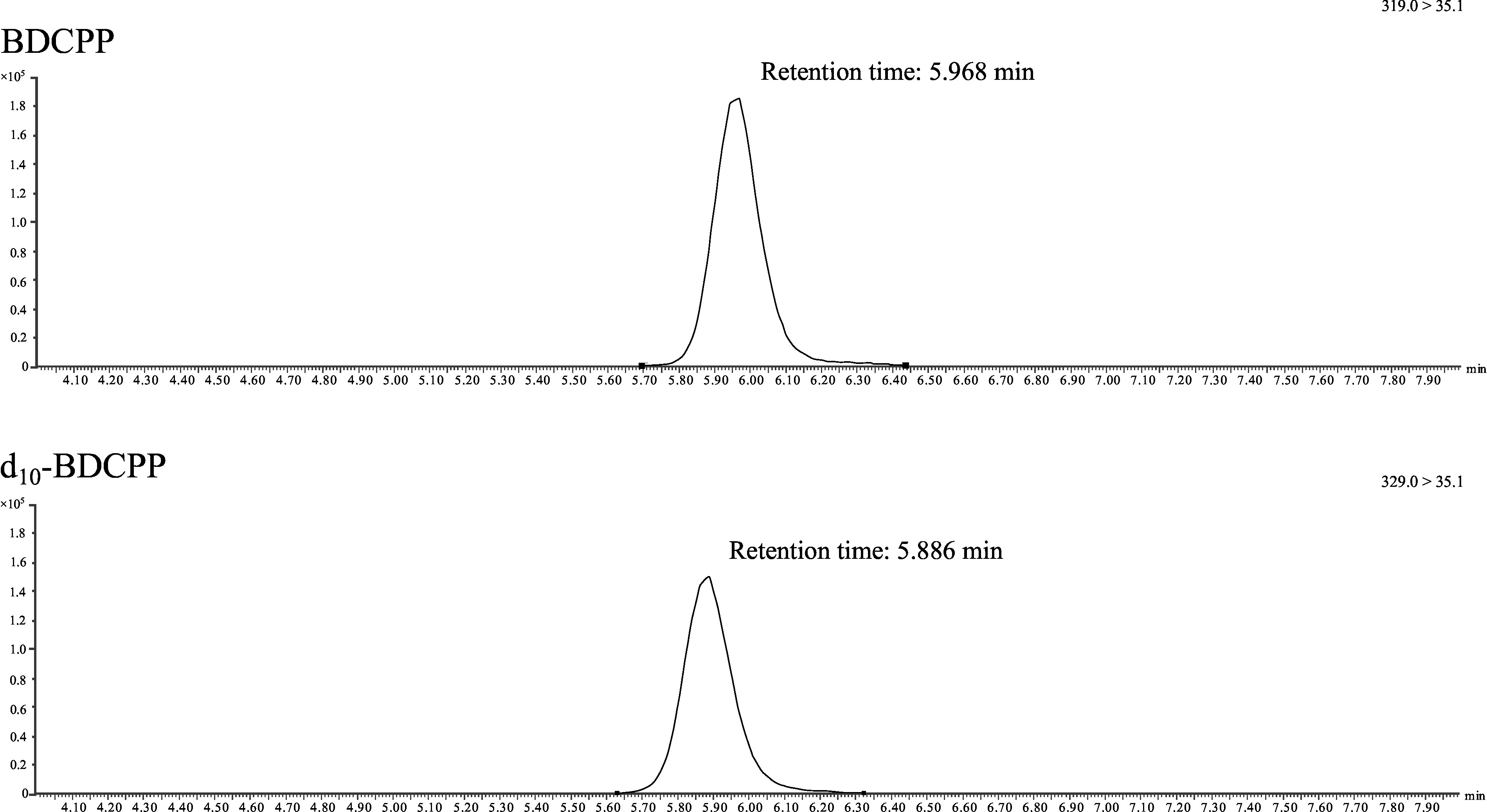
Chromatogram of a standard spiked with 90 μg BDCPP as well as 90 μg d_10_‑BDCPP per litre

**Fig.3:**
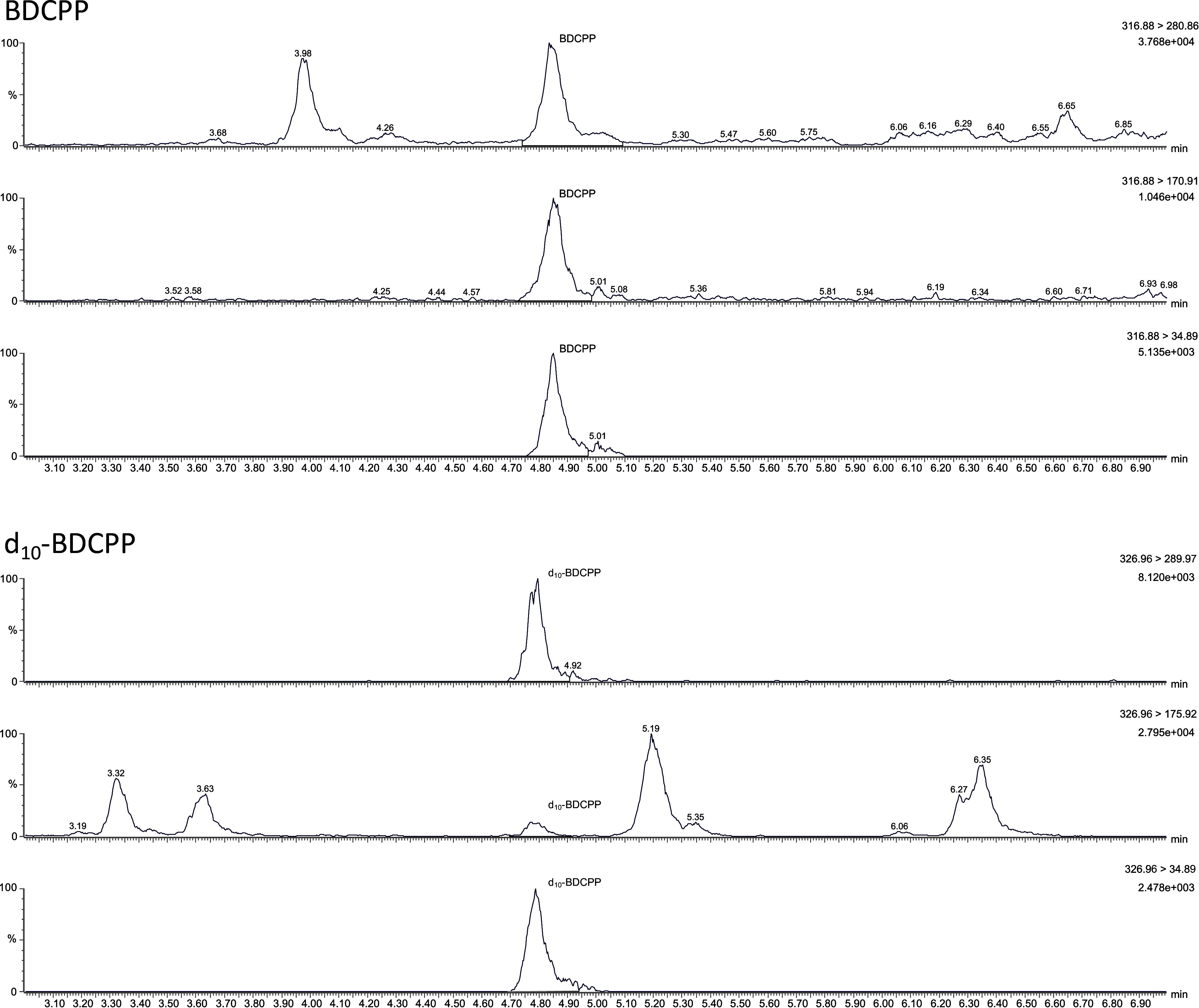
Chromatogram of a pooled-urine sample spiked with 20 μg BDCPP per litre as well as 10 µg d_10_-BDCPP per litre

Figure 2 shows a representative chromatogram of a standard spiked with 90 μg BDCPP as well as 90 μg d_10_‑BDCPP per litre (method development). Figure 3 shows a representative chromatogram of a pooled-urine sample spiked with 20 μg BDCPP as well as 10 μg d_10_‑BDCPP per litre (external verification).

## 8 Calibration

The calibration solutions are prepared as described in Section 4.5, processed analogously to the urine samples without the further addition of ISTD (see Section 5.2), and analysed. The calibration curve is generated by plotting the peak areas of the analyte and ISTD, respectively, against the spiked concentrations. The calibration curve of d_10_‑BDCPP is used to calculate the recovery of the ISTD (see Section 9).

**Fig.4:**
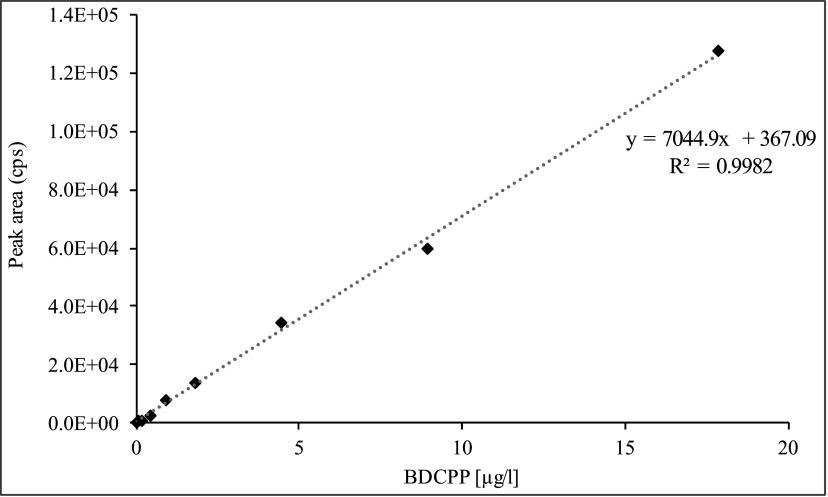
Calibration curve for the determination of BDCPP in urine samples of the non-occupationally exposed general population

The calibration curve of BDCPP is linear up to 1000 μg/l. A concentration range of 0.1–100 μg/l should be sufficient for the quantitation of occupational exposure. For the quantification of background exposure in the non-occupationally exposed general population, a calibration range from 0.1–20 μg/l is applied (see Figure 4).

## 9 Calculation of the analytical results

The slope of the calibration curve is calculated by linear regression and applied for quantification. To this end, the intercept-corrected peak area of the blank value is subtracted from the intercept-corrected peak area of BDCPP in the sample and, with regard to the slope of the calibration curve, the analyte concentration is calculated in μg/l. The concentration is further corrected with respect to the different volumes of the calibration standards and the measured urine samples.

The verifiers of the method used calibration standards prepared in urine which, at first, only contained BDCPP. Of these standards, aliquots of 5 ml each were processed analogously to the urine samples and, as a result, maintained a constant concentration of 10 μg ISTD per litre of urine. 

**Fig.5:**
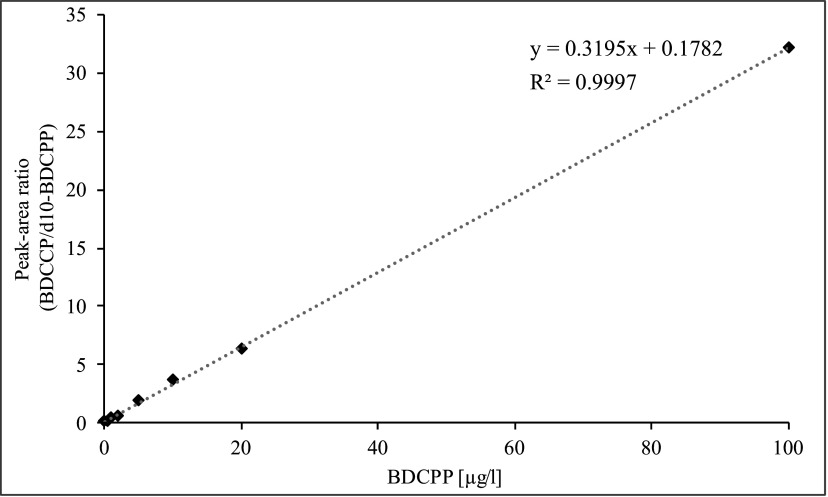
Calibration curve for the determination of BDCPP in urine (external verification)

During external verification, the calibration curve was generated by plotting the peak-area ratios of analyte and ISTD against the corresponding BDCPP concentration (see Figure 5). If blank values arose, they were subtracted from all measurement points. To calculate the BDCPP concentration in a urine sample to be measured, the peak area determined for BDCPP was divided by the peak area of the deuterated ISTD. The analytical result was calculated in μg/l by inserting the quotient thus obtained into the calibration function.

This approach is mathematically identical to that of the method developers.

## 10 Standardisation and quality control

Quality assurance of the analytical results is carried out as stipulated in the guidelines of the *Bundesärzte­kammer*(German Medical Association) and in a general chapter published by the Commission (Bader et al. 2010; Bundesärztekammer 2014).

For quality assurance of the individual analytical runs, at least three urines with known analyte concentrations are processed and analysed parallel to the samples. Since no control materials are currently commercially available for BDCPP, these must be prepared in the in-house laboratory. To this end, pooled urine from persons not occupationally exposed to TDCPP is used and, for example, spiked with BDCPP at concentrations of 1.0 μg, 10 μg, or 100 μg per litre.

At the same time, blanks (ultra-pure water) and reagent blanks are included in each analytical run in order to recognise any potential interferences from reagents, matrix components, or the ISTD.

## 11 Evaluation of the method

The reliability of this method was confirmed by comprehensive validation as well as by replication and verification of the method in a second, independent laboratory.

### 11.1 Precision

#### Within-day precision

To determine within‑day precision, a pooled-urine sample was spiked with 0.9 μg, 9 μg, or 90 μg BDCPP/l, processed eight times in parallel, and analysed. The precision data calculated from the measurement results are shown in Table 6.

**Tab.6:** Within-day precision for the determination of BDCPP in urine (n = 8)

Analyte	Spiked concentration [μg/l]	Standard deviation (rel.) *s_w_* [%]	Prognostic range *u* [%]
BDCPP	0.9	6.5	15.4
	9	3.1	7.3
	90	5.5	13.0

#### Day-to-day precision

Day-to-day precision was determined using the same material as was used for within-day precision. The samples were processed and analysed on eight days. The precision data thus obtained are given in Table 7.

**Tab.7:** Day-to-day precision for the determination of BDCPP in urine (n = 8)

Analyte	Spiked concentration [μg/l]	Standard deviation (rel.) *s_w_* [%]	Prognostic range *u* [%]
BDCPP	0.9	8.5	20.1
	9	8.7	20.6
	90	5.5	13.0

### 11.2 Accuracy

The relative recovery was determined using the day-to-day precision data by comparing the measured concentrations with the spiked concentrations. The results are shown in Table 8.

**Tab.8:** Relative recovery for the determination of BDCPP in urine (n = 8)

Analyte	Spiked concentration [μg/l]	Mean relative recovery *r* [%]
BDCPP	0.9	93.6
	9	99.5
	90	100.5

### 11.3 Influence of various urine matrices

The influence of various urine matrices on precision and recovery was tested using ten individual urine samples. The creatinine concentrations of the urine samples ranged from 1.3 mmol to 22.7 mmol per litre. The samples were spiked with 90 μg BDCPP per litre of urine and subsequently processed and analysed.

The standard deviation and the mean relative recovery, calculated from the measurement results, are given in Table 9. It is evident that the individual urine matrix does not have any influence on the precision and accuracy of the analytical results.

**Tab.9:** Influence of individual urine matrices on the determination of BDCPP in urine (n = 10)

Analyte	Spiked concentration [μg/l]	Standard deviation (rel.) ***s***_w_ [%]	Relative recovery *r* [%]
BDCPP	90.0	5.8	92.3

### 11.4 Limits of detection and quantitation

The limits of detection and quantitation for the determination of BDCPP in urine were calculated according to NEN 7777+C1 (NEN 2012) (multiple determinations of one laboratory sample). To this end, eight urine samples were spiked with 0.9 μg BDCPP per litre of urine, processed, and analysed on one day. These processed samples were further analysed on eight different days. The calculated BDCPP concentrations were in the range of 0.73–1.00 μg per litre; the average of 0.84 μg BDCPP/l was used for further calculations.

The detection limit was calculated as three times the standard deviation of the measured mean, whereas the quantitation limit was calculated as ten times this value. Table 10 shows the respective values for the determination of BDCPP in urine.

**Tab.10:** Limits of detection and quantitation for the determination of BDCPP in urine

Analyte	Detection limit [μg/l]	Quantitation limit [μg/l]
BDCPP	0.06	0.2

### 11.5 Stability of BDCPP in urine and in the extracts

Storage tests were performed to assess the storage stability of BDCPP in the urine samples as well as in the extracts. To this end, pooled urine was aliquoted and spiked with 0.9 μg, 9 μg, and 90 μg BDCPP per litre and stored in the dark at various temperatures (−21 °C, 3 °C, and 21 °C). The samples were processed and analysed after 0, 24, 48, 72, and 168 hours. Moreover, SPE extracts from directly processed samples were stored at −21 °C, 3 °C, and 21 °C and analysed after the aforementioned storage times.

Relative recoveries in the range of 91–104% confirmed that BDCPP is stable under the tested conditions.

### 11.6 Sources of error

Potential carryover effects were tested by alternately measuring processed samples of spiked (90 μg BDCPP/l urine) and unspiked urine (n = 6 in each case). No significant carryover effects were observed, as BDCPP was not detected in any of the six unspiked urine samples.

For method development, a LC‑MS/MS system with an Agilent multimode source was applied, allowing for simultaneous ESI and APCI ionisation. The sensitivity of the mixed-mode setting is similar or better when compared to dedicated ion sources (Fischer and Perkins 2005).

The instrumentation of the method verifiers did not have a multimode source, such that the verifiers had to choose between ESI or APCI mode. As the ionisation rate was better for ESI ionisation than for APCI ionisation, the ESI mode was applied during external verification. Using only ESI, the verifiers of the method could confirm the mass transitions reported by the method developers, but other mass transitions turned out to be more pronounced. The alternative mass transitions used during external verification are given in Table 11 alongside the retention times and further MS/MS parameters. 

The LOQ determined by the verifiers of the method (3.0 μg/l) was higher than that reported by the developers (0.2 μg/l), and was therefore too high for the determination of most background exposure levels. As a result, simultaneous ESI and APCI ionisation is essential for the sensitive quantitation of BDCPP background levels using this method.

**Tab.11:** Retention times, mass transitions, and MS/MS parameters for the determination of BDCPP in urine (external verification)

Substance	Retention time [min]	Precursor ion (***m/z***)	Product ion (***m/z***)	Collision energy [V]	Cone voltage [V]
BDCPP	4.6	316.9	280.9^a)^	6	14
		316.9	170.9^b)^	8	14
		316.9	34.9^b)^	6	14
d_10_‑BDCPP	4.5	327.0	290.0^a)^	6	8
		327.0	175.9^b)^	8	8
		327.0	34.9^b)^	6	8

a) Quantifier

b) Qualifier

## 12 Discussion of the method

The LC‑MS/MS method described herein is based on basic procedural steps from published methods for the quantitation of BDCPP in urine (Carignan et al. 2013; Cooper et al. 2011; Dodson et al. 2014; Hoffman et al. 2014; Meeker et al. 2013 b; Van den Eede et al. 2015). The combination of APCI and ESI, operating in negative ionisation mode with the simultaneous use of Multiple Reaction Monitoring, allowed for the selective, sensitive, and robust quantitation of BDCPP in urine.

With within-day precision of 3.1–6.5% and day-to-day precision of 5.5–8.7%, the reliability criteria of the method can be described as excellent. The accuracy of the method was confirmed by high recovery rates in both pooled urine and individual urines after spiking. Moreover, the selectivity of the LC‑MS/MS method is very high. Significant interfering peaks were not observed for the mass transitions selected for quantification.

The method therefore enables the sensitive and valid quantitation of BDCPP in urine. The quantitation limit of 0.2 μg BDCPP per litre of urine is sufficient for application in the field of occupational medicine and is partially sufficient for the quantitation of background levels in the general population. In order to achieve this low limit of quantitation, a multimode source must be applied which allows for simultaneous APCI and ESI.

**Instruments used **LC‑MS/MS system connected to a triple-quadrupole mass-spectrometer with a multimode source (Agilent 1200 series and Agilent 6460, Agilent Technologies, Inc., Santa Clara, CA, USA); reversed-phase column, Kinetex^®^core-shell silica (2.6 μm biphenyl 100 Å, 100 × 2.1 mm) (No. 00D‑4622‑AN, Phenomenex Inc., Torrance, CA, USA) with a SecurityGuard precolumn (No. AJ0‑9209, Phenomenex Inc., Torrance, CA, USA)

## Notes

The method originally developed (Krystek et al. 2019) was part of a 10‑year project on the advancement of human biomonitoring in Germany, which was based on a cooperation agreed in 2010 between the Federal Ministry of the Environment, Nature Conservation, and Nuclear Safety (BMU) and the Verband der chemischen Industrie e. V. (German Chemical Industry Association, VCI).
